# Maternal and Infant Determinants of Zinc Status and Zinc’s Association with Anthropometry in 3-Month-Old Bangladeshi Infants †

**DOI:** 10.3390/nu17213393

**Published:** 2025-10-29

**Authors:** Ximing Ge, Katherine K. Stephenson, Lee S.-F. Wu, Sarah Baker, Hasmot Ali, Saijuddin Shaikh, Keith P. West, Parul Christian, Kerry J. Schulze

**Affiliations:** 1 Center for Human Nutrition, Department of International Health, Johns Hopkins Bloomberg School of Public Health, Baltimore, MD 21205, USA; xge7@jhu.edu (X.G.); kstephe2@jhmi.edu (K.K.S.); lwu2@jhu.edu (L.S.-F.W.); sbaker52@jhu.edu (S.B.); kwest1@jhu.edu (K.P.W.J.); pchrist1@jhu.edu (P.C.); 2 The JiVitA Project, Johns Hopkins Bloomberg School of Public Health, Gaibandha 5700, Bangladesh; hasmot.jivita@gmail.com (H.A.); saijuddin.shaikh@sas.org.in (S.S.)

**Keywords:** zinc, infant, mothers, human milk, multiple micronutrient supplementation

## Abstract

Background/Objectives: Zinc deficiency remains a public health concern in South Asia but is rarely studied through gestation to infancy. Methods: We identified maternal and infant factors related to plasma zinc of 3 mo old Bangladeshi infants (*n* = 317) in the context of a trial of a daily antenatal to 3 mo postpartum multiple micronutrient supplementation (MMS) with 15 vitamins and minerals, including 12 mg zinc, versus iron–folic acid (IFA). Factors explored included maternal age, parity, and plasma zinc in early (pre-supplementation) and late pregnancy, at 3 months postpartum, and in milk; cord blood zinc (*n* = 83); birth outcomes; and infant feeding and biomarkers. Consequently, infant zinc was explored with 3 mo anthropometry and growth rates. Results: Mean ± SD infant plasma zinc was 15.63 ± 6.65 µmol/L, with 10.1% deficiency (<9.9 µmol/L). In adjusted analyses, infant zinc was positively associated with maternal age [20–30 years +0.11 µmol/L (*p* = 0.018) and ≥30 years +0.28 µmol/L (*p* = 0.003) relative to <20 years], maternal early pregnancy zinc (+0.01 µmol/L per 1 µmol/L maternal zinc, *p* = 0.011), and infant ferritin (+0.001 µmol/L per 1 µg/L, *p* = 0.007); conversely, infant zinc was −0.13 µmol/L lower (*p* = 0.013) with maternal parity ≥2 versus 0–1 and with partial versus exclusive breastfeeding (−0.15 µmol/L, *p* = 0.038). Relationships with MMS, maternal later pregnancy, postpartum, milk, and cord blood zinc were absent. Length-for-age (+0.02 per µmol/L, *p* = 0.047) but not weight-for-length Z-scores at 3 months were associated with infant zinc. Conclusions: Thus, infant zinc was associated with pre- but not post-MMS maternal zinc, age and parity, feeding style, and infant iron status. Infant length but not weight was associated with plasma zinc.

## 1. Introduction

Zinc is an essential trace mineral, acting as a cofactor for many metalloenzymes and playing a vital role in cellular growth, immune function, and the synthesis of DNA and RNA [1,2]. Zinc is particularly crucial during the first 1000 days of life, spanning from conception through the first two years, playing a vital role in fetal development, infant growth, immune function, and early cognitive development [3,4,5]. In children, zinc deficiency has been linked to increased susceptibility to infectious diseases, impaired growth, and higher mortality [6]. Zinc supplementation reduces the duration and severity of diarrhea, a leading cause of death in children under five, and produces a modest improvement in linear growth among children aged 6 months to 12 years [7].

Despite its importance, zinc deficiency remains a significant public health concern, particularly in developing countries where plant-based diets with low zinc bioavailability are common [8]. The prevalence of zinc deficiency is notably high in South Asia [9,10]. In Bangladesh, nationally representative assessments found serum zinc deficiency in 44.6% of preschool children (6–59 months) and 57.3% of non-pregnant, non-lactating women (15–49 years) in the 2011–12 National Micronutrient Survey [11]. Zinc status in populations is most commonly assessed by serum or plasma zinc concentrations, with cutoffs originally derived from representative data from the US and later adopted by the International Zinc Nutrition Consultative Group (IZiNCG) as international recommendations [12,13]. However, an important limitation is the absence of validated cutoffs for infants—particularly those younger than six months, when exclusive breastfeeding is recommended—a group generally excluded from studies/surveys, leaving their zinc status poorly characterized.

The dynamics of zinc transfer from mother to infant in pregnancy and lactation are complex. During pregnancy, maternal zinc absorption is increased to allow for the transfer of zinc to the fetus, which depends on the sufficiency of zinc in maternal circulation [14]. The placenta regulates zinc transfer to the fetus over the course of pregnancy, with studies suggesting that placental zinc transport increases as gestation progresses [15]. Postnatally, the mammary gland employs a different set of zinc transporters to secrete zinc into milk [16]. Postpartum, zinc concentrations in breast milk undergo significant changes over the course of lactation, with zinc being highest in colostrum and then decreasing in the first few days after birth and reaching the lowest levels between 7 and 12 months of lactation [17].

Evidence on circulating zinc concentrations in early infancy is limited, but studies from Bangladesh provide important insights. In rural Bangladesh, plasma zinc measured at 6 months showed higher concentrations in infants exclusively breastfed for 4–6 months versus in those <4 months, with over half of all infants below the cutoff for deficiency [18]. In Dhaka urban slums, another trial where zinc supplementation was given via oral liquid (5ml) from 1 month of age showed that mean serum zinc was lower in the placebo group at 6 months [19]. Preterm and low birth weight (LBW) infants begin life with reduced zinc reserves, as shown by lower cord blood zinc concentrations compared to term infants [20,21]. Evidence directly linking circulating zinc of early infancy is limited, and overall there remains a lack of research on other maternal and infant factors that may influence infant zinc.

In this study, infant plasma zinc concentrations were assessed among 3-month-old infants from rural Bangladesh in the context of an antenatal trial of multiple micronutrient supplementation (MMS), which included 15 micronutrients, including iron and folic acid and 12 mg/d of zinc, compared to iron–folic acid (IFA) alone as standard of care, provided daily from early pregnancy through 3 months postpartum. The primary objective was to identify factors associated with infant plasma zinc at 3 months of age from among variables, including the intervention, maternal plasma zinc in early and late pregnancy and at 3 months postpartum, maternal milk zinc concentrations, infant feeding regimens, and other maternal and infant characteristics. In addition to potentially shedding light on aspects of zinc transfer from mother to infant during pregnancy and lactation, we suggest that understanding both modifiable and unmodifiable factors associated with infant zinc could help identify ways to improve zinc status during infancy. Furthermore, we sought to determine whether infant zinc was in turn associated with infant growth parameters to ascertain potential consequences of poorer zinc status in early infancy.

## 2. Methods

### 2.1. Study Design, Population, and Field Procedures

The JiVitA-3 double-blinded, cluster-randomized trial was conducted in 18 unions in Gaibandha District and 1 neighboring union in Rangpur District of northern Bangladesh, divided into 596 sectors [22]. Recruitment was conducted by a home-based 5-weekly pregnancy surveillance system, with an initial enrollment of over 44,000 women giving birth to 14,142 infants in the IFA group and 14,374 infants in the MMS group. The trial showed that the MMS supplement, extended gestational duration and reduced preterm birth and low birth weight, resulting in significantly larger babies based on birth weight, length, and circumferential measures [22].

### 2.2. Substudy Biospecimen Collection

In a more intensive “substudy” that was nested in the main trial and took place in 64 pre-determined sectors, initially enrolling over 2000 participants, maternal venous blood was collected at baseline and 32 weeks of gestation in participants’ homes by highly trained female phlebotomists [23]. Maternal and infant blood samples and maternal milk were also collected at 3 months postpartum among substudy participants in a similar manner. In a further nested study, cord blood was collected for a limited period of time among participants in 31 sectors (*n* = 333 newborns) [24]. Milk was collected in spot samples from a single breast, most recently not used to feed the infant, by self-collection during home visits; infant blood (300 µL) was collected from heel sticks using Saf-T-fill (Ram Scientific, Nashville, TN, USA) devices. Blood was separated by centrifugation in a field laboratory into plasma, which was frozen on liquid nitrogen before being shipped to Johns Hopkins Bloomberg School of Public Health and frozen at −80 °C for later use. Milk was mixed upon receipt in the field laboratory and dispensed into multiple 2 mL aliquots and also frozen in liquid nitrogen storage containers until being shipped and stored at −80 °C. The procedures and biochemical data from the pregnancy and birth visits have been described previously in detail for a cohort of 1526 women with serial biospecimen collections that formed the sampling frame for the postpartum analysis described in this paper [23].

The JiVitA-3 Iron-Folic Acid versus Multiple Micronutrients in Milk for Infants Study (JIMMI Study): Because we could not measure all micronutrient biomarkers of interest [zinc, vitamin A and E, vitamin D, ferritin, alpha-1 acid glycoprotein (AGP), vitamin B12, and untargeted metabolomics] in the limited amount of plasma obtained from infants, subsets of overlapping samples were assigned to various assays to achieve balance by maternal intervention status, infant sex, and breastfeeding status (exclusive, predominant, and partial breastfeeding). Here, data were analyzed for 317 infants where corresponding maternal zinc data were complete for early and late pregnancy and postpartum time points. Cord blood zinc was available for a subset of these cases (*n* = 83).

### 2.3. Laboratory Analyses

The maternal plasma zinc concentration in pregnancy [23] and infant cord blood plasma zinc [24] had previously been analyzed using graphite furnace atomic absorption spectroscopy (AAnalyst 800; PerkinElmer, Shelton, CT, USA), validated against National Institute of Standards and Technology Standard Reference Material 1598a (NIST SRM, Gaithersburg, MD, USA), Inorganic Constituents in Animal Serum [25] and Trace Elements Serums L1 and L2 (Seronorm; SERO, Billingstad, Norway). The infant 3-month ferritin concentration was assessed by commercial enzyme-linked immunoassay kits (ELISA; Sigma-Aldrich, St. Louis, MO, USA). Infant 3-month AGP was examined using a radial immunodiffusion kit (Kent Laboratories, Bellingham, WA, USA), serving as an indicator for inflammation. Assays for infant ferritin and AGP were conducted with commercially prepared quality control materials.

Infant plasma zinc was analyzed using graphite furnace atomic absorption spectroscopy (AAnalyst 800; PerkinElmer) using 16 µL plasma diluted to 1:125 in water confirmed to be zinc-free prior to each sample run. Calibration curves were generated daily using dilutions of Seronorm controls and tested for validity per run with NIST SRM 1598a. For quality control purposes, NIST, Seronorms, and pooled plasma with a known zinc concentration were analyzed and monitored every 15 samples.

Maternal 3-month postpartum zinc, along with other trace minerals, was analyzed using 100 µL of plasma by inductively coupled plasma mass spectrometry (7850 ICP-MS; Agilent, Santa Clara, CA, USA). Plasma was diluted with 1.4 mL of basic diluent [26], with internal standard (100 ppm of Li, Sc, Ge, Rh, In, Tb, Lu, and Bi in 10% HNO3; Agilent) added, and 2.3 mL of LC/MS water (Fisher Scientific, Fair Lawn, NJ, USA), for a total volume of 4 mL. Basic diluent was made up using 4% 1-butanol, 0.15% Triton X-100, 0.15% Ethylenediaminetetraacetic acid, and 1% ammonium hydroxide in LC/MS water (Fisher Scientific). The zinc standard (Agilent) was used to generate the standard curve, with Seronorms at two levels, NIST, and pooled plasma, with a known zinc concentration run to ensure assay validity every 10 samples. The AAS and ICP-MS approaches were cross-validated for zinc analysis, with the relation described by the equation ICP-MS = 0.96 * AAS − 0.69 in µmol/L. No adjustment was made to reconcile methods.

Human milk was also analyzed by inductively coupled plasma mass spectrometry (7850 ICP-MS; Agilent) using an acid digestion [27]. In sample digestion tubes (Xpress Vessels; CEM, NC, USA), 200 µL breastmilk, 300 µL ultrapure nitric acid (67–70%; J.T.Baker, Phillipsburg, NJ, USA), and 2.5 mL hydrogen peroxide (30%; J.T.Baker) were mixed. Samples were combined with nitric acid and left in the fume hood for a pre-digest of 15 min prior to adding hydrogen peroxide. Then, the mixture was digested at 200 °C for 15 min using a microwave digester (Mars 6; CEM). Then, 1.3 mL of cooled digested mixture was added to 1.3 mL of LC/MS water (Fisher Scientific) containing internal standard (100 ppm of Li, Sc, Ge, Rh, In, Tb, Lu, Bi in 10% HNO3; Agilent). NIST SRM 1954 [28], NIST SRM 1849a [29], pooled breastmilk with a known zinc concentration, and a blank were prepared in the same manner for quality control purposes. An inorganic zinc standard (Agilent) was used to generate the standard curve.

### 2.4. Statistical Analysis

Because of our interest in infant growth outcomes, which are known to differ by infant sex, population characteristics were described for all infants and stratified and compared by infant sex—including maternal characteristics and infant characteristics at birth and 3 months—using chi-square tests for categorical variables and Welch’s *t*-tests for continuous variables. Additionally, these characteristics were compared between substudy participants and the larger trial population to assess the representativeness of the substudy sample within the broader community of JiVitA-3 trial participants. Characteristics were also assessed by intervention group to ensure balance was retained in this subset of participants.

Infant zinc distributions for this subset of JiVitA-3 participants are shown side-by-side with zinc distributions of maternal zinc in the first and third trimesters, in maternal plasma and milk at 3 months postpartum, and in infant cord blood to show patterns in concentrations across the reproductive event. This is to provide a visual summary of all the zinc data types and time points that were explored in relation to infant zinc.

To explore determinants of infant plasma zinc, generalized estimating equations (GEEs) were used to adjust for the design effect related to the cluster-randomization. Observations with standardized residuals exceeding ±2.5 were excluded during model fitting to address outlier influence, with this exclusion applied independently for each regression analysis. Breast milk zinc was log-transformed prior to analysis to address skewness; other zinc distributions were not as skewed, and the arithmetic scale was retained. Zinc deficiency was defined using established cutpoints from the IZiNCG guidelines: 9.9 µmol/L for infants, 8.6 µmol/L for early pregnancy, 7.6 µmol/L for late pregnancy, and 10.1 µmol/L for postpartum women [12,13]. A wide range of other variables with plausible associations with infant zinc concentrations were selected for evaluation. These variables were first analyzed through separate models developed for either maternal or infant characteristics to narrow down candidate factors with infant zinc as the outcome. Maternal factors examined, aside from study intervention and maternal plasma and milk zinc, included age, parity, maternal baseline zinc, and intervention status. Other variables initially explored but not retained in the final model included maternal BMI at different stages, gestational weight gain, literacy status, socioeconomic status, and seasonality. Multicollinearity diagnostics between maternal age and parity were tested. Infant factors comprised age, sex, gestational age at birth, breastfeeding status at 3 months postpartum (exclusive, predominant, partial), and available biomarkers, including ferritin and AGP. Core variables (maternal zinc, intervention status, infant age, and breast milk zinc) were retained in final models regardless of significance. Also tested were gestational age at birth, infant anthropometry (length and weight), and, in the subset where available, cord blood zinc—none of which were retained as important determinants of infant zinc. The final combined multivariate model demonstrated better goodness-of-fit statistics compared to separate maternal and infant characteristic models.

Anthropometric outcomes were assessed using the World Health Organization (WHO) Child Growth Standards [30]. Length was evaluated as length-for-age z-scores (LAZs), proportionality as weight-for-length z-scores (WLZs), and total weight as weight-for-age z-scores (WAZs) [30]. The rate of weight gain was calculated as weight at 3 months minus weight at birth (in grams), divided by infant age in days. The rate of length gain was calculated as length at 3 months minus length at birth (in centimeters), divided by infant age in months (30 days).

GEE models examined associations of infant anthropometry at 3 months and growth from birth to 3 months as outcomes in relation to infant zinc at 3 months as the primary independent variable, adjusted for infant age, sex (not for the Z-score model), breastfeeding status, gestational age, and corresponding birth anthropometrics (length and weight). These covariates were identified through model selection or chosen a priori because of known or hypothesized relationships between these maternal and infant characteristics and infant growth outcomes.

Data was exported to STATA (StataCorp LLC, College Station, TX, USA) 17.0 BE-basic Edition for analysis, and *p* < 0.05 was considered statistically significant.

## 3. Results

### 3.1. Study Sample Characteristics

Features of participants in this analysis were similar to those from the larger JIMMI study (Supplementary Table S1), suggesting reasonable representation of the JiVitA-3 field site in these analyses. In Table 1, maternal and infant characteristics are shown for all contributors to this analysis and by infant sex. At enrollment, mothers were young, predominantly in their twenties, with roughly one-third nulliparous and over half literate. Participants were generally short in stature and had low early-pregnancy weight. Maternal plasma and milk zinc concentrations did not differ by infant sex or intervention. The proportion of male and female infants was balanced.

Gestational age at birth and birth outcomes did not differ by infant sex, with a high prevalence of LBW (42.0%), small-for-gestational age (SGA; 67.4%), and preterm birth (11.7%) that was less prevalent. At 3 months, the mean WAZ (−1.4 ± 1.1), LAZ (−1.3 ± 1.0), and weight gain (28.5 ± 6.1 g/day) reflected some gains against the WHO standard, yet small infant size remained throughout early infancy. Most infants (83.0%) at 3 months of age were exclusively breastfed. Mean infant ferritin was 147.2 ± 96.8 μg/L, and 32% of infants had inflammation (AGP > 1 g/L). Mean infant plasma cord blood concentrations were nearly 15 µg/L, similar to zinc concentrations of 15.3 ± 6.4 μmol/L at 3 months, with no significant difference between male and female infants.

However, significant sex differences were observed in infant anthropometry. Male infants had greater birth length and maintained higher anthropometric measures at 3 months of age, with a greater weight, length, and rate of length gain compared to female infants (*p* < 0.05); meanwhile, corresponding anthropometric z-scores (WAZ, LAZ, and WLZ) did not differ by sex. Additionally, female infants had significantly higher ferritin levels than male infants (*p* = 0.02), and breastfeeding status differed between sexes (*p* = 0.007), with exclusive breastfeeding more common among female infants.

### 3.2. Distribution of Zinc Concentrations

**Figure 1:**
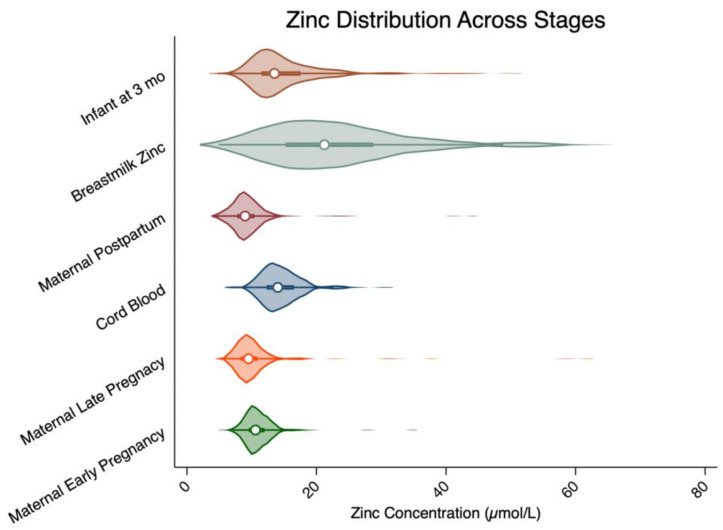
The distribution of zinc concentration in early pregnancy, late pregnancy, cord blood, infant, breast milk and maternal postpartum ^1,2^. ^1^ Infants at 3 months: *n* = 317; breastmilk zinc: *n* = 268; maternal postpartum: *n* = 315; cord blood: *n* = 83; maternal late pregnancy: *n* = 315; maternal early pregnancy: *n* = 316. For better visualization, breastmilk zinc concentrations > 80 µmol/L were truncated (*n* = 5). ^2^ White circles of the violin plots indicate the distribution medians, with the thick bar representing the interquartile ranges (IQRs; 25th–75th percentile).

Figure 1 illustrates the distribution of plasma zinc concentrations across the reproductive cycle, from early pregnancy through 3 months postpartum. Based on cutpoints of 9.9, 8.6, 7.6, and 10.1 µmol/L for infants, early pregnancy, late pregnancy, and postpartum, respectively [12,13], the prevalence of zinc deficiency was 10.1% in infants, 8.8% in mothers in early pregnancy, 13.2% in late pregnancy, and markedly higher at 70.0% in postpartum women. Because no postpartum-specific zinc cutoff has been established, we applied the nonpregnant adult female threshold, which is considerably higher than recommended cutoffs during pregnancy; consequently, postpartum deficiency estimates are not directly comparable with pregnancy estimates. Cord blood zinc was higher and distributed more symmetrically than maternal zinc, and infant zinc at 3 months also exceeded maternal concentrations. Breast milk showed the highest median and the widest range in concentration, with prominent high outliers. Together, these cross-sectional plots highlight unique distributions of maternal to cord blood to infant zinc.

### 3.3. Determinants of Infant Plasma Zinc

The results of unadjusted and adjusted regression analyses to ascertain factors associated with infant zinc are shown in Table 2. Among maternal factors, a tendency toward higher infant zinc with increasing maternal age category that was present in the unadjusted model became increasingly apparent in the adjusted model, such that infant zinc averaged 0.11 µmol/L higher among 20–30 year old mothers (*p* = 0.018) and 0.28 µmol/L higher among women >30 years (*p* = 0.003) compared to values for women <20 years of age. Conversely, an association of lower zinc in the highest parity category was observed in the adjusted model as infant zinc was 0.13 µmol/L lower among women with parity ≥2 (*p* = 0.013) when age was accounted for. Early pregnancy maternal plasma zinc concentrations pre-supplementation were positively associated with infant zinc in both unadjusted (β = 0.020, *p* = 0.001) and adjusted models (β = 0.012, *p* = 0.011). However, notably, the MMS intervention, maternal plasma zinc at late pregnancy and 3 months postpartum, and cord blood zinc were not significantly associated with infant zinc when tested in separate adjusted models (Table 3).

Among infant factors, age and sex were retained as predetermined covariates based on biological plausibility. Partial breastfeeding was associated with 0.15 µmol/L lower infant zinc than exclusive breastfeeding (*p* = 0.038), with the effect of predominant breastfeeding intermediary but not significantly different from zinc during exclusive breastfeeding, though suggesting a trend of poorer infant zinc with increasingly less ideal breastfeeding behavior. However, breast milk zinc concentrations were not themselves associated with infant plasma zinc. Both infant ferritin (*p* = 0.007 in both adjusted and unadjusted models) and AGP (*p* = 0.004 unadjusted; *p* = 0.160 adjusted) were included as iron status and inflammation biomarkers, with infant zinc averaging 0.001 µmol/L higher per 1 µg/L ferritin increment in the adjusted model.

### 3.4. Relationship of Infant 3 Mo Anthropometry to Infant Zinc

Associations of infant anthropometric indicators at 3 months with infant plasma zinc are presented in Table 4. Among the growth parameters examined, 3 mo LAZ demonstrated a positive association with infant plasma zinc that persisted in both the unadjusted and adjusted models. This relationship remained significant after controlling for breastfeeding status, gestational age, and intervention, indicating that each 1 µmol/L increase in infant zinc was associated with nearly a 0.02 higher LAZ. The WAZ was not associated with infant plasma zinc in either model; however, an inverse association of the WLZ with infant zinc that approached statistical significance was present in the adjusted model, with an average 0.014 lower WLZ per 1 µmol/L increment in infant zinc (*p* = 0.06).

Associations of linear and ponderal growth rates from birth to 3 months with infant zinc are shown in Table 4. Gains in length were not associated with infant plasma zinc in the unadjusted model, but a marginal signal emerged in the adjusted model of a 0.006 cm/mo (95% CI: −0.001, 0.014; *p* = 0.095) increase per 1 µmol/L increment in plasma zinc. There was no association between the rate of weight gain and infant plasma zinc.

## 4. Discussion

This study demonstrated associations of infant plasma zinc concentrations at 3 months postpartum in rural Bangladesh with both maternal characteristics (early pregnancy zinc, age, and parity), infant characteristics (ferritin and AGP), and breastfeeding status. Maternal zinc status in early pregnancy—but not in late pregnancy, at 3 months postpartum, or in breast milk—was associated with infant zinc status. Additionally, daily maternal zinc supplementation with other micronutrients through 3 months postpartum did not impact infant zinc status. In turn, infant plasma zinc was positively associated with length but not weight, with a modest inverse association with weight-for-length and positive association with a linear growth rate from birth to 3 months. Taken together, these findings suggest that an understanding of factors related to infant zinc concentrations—whether modifiable or not—could contribute to means to improve infant zinc status and its associated outcomes.

Maternal age was a significant determinant of infant zinc concentrations. Infant zinc concentrations increased progressively with maternal age, from the lowest concentrations among infants of mothers <20 years of age to the highest in those born to mothers 30 years and older. This pattern suggests that younger mothers, particularly adolescents, might be less able to adequately meet the zinc demands of pregnancy and early infant development [14]. The relationship likely reflects competing zinc demands between maternal growth and fetal requirements during adolescent pregnancy. The socioeconomic implications of early marriage and adolescent pregnancy in Bangladesh may further compound these biological constraints through reduced access to zinc-rich foods [31,32].

Because age and parity are positively associated, an inverse association of parity with infant zinc was unexpected. Our findings are consistent with an independent inverse effect of parity on infant plasma zinc for a given maternal age. We speculate that the inverse relationship between maternal parity and infant zinc status could reflect zinc depletion with successive pregnancies. Limited research has examined the relationship between parity and infant zinc concentrations. While no studies have directly investigated the association with infant zinc concentrations, two studies have examined maternal zinc concentrations in relation to parity [28,29], though findings are inconsistent. These limited studies highlight the substantial gap in understanding how reproductive history affects trace element status of infants.

The positive association between maternal early pregnancy zinc and infant zinc concentrations represents another independent factor affecting infant zinc status. This finding highlights the possibility that infant zinc status could be improved by improving maternal status preconceptionally, potentially through zinc supplementation prior to pregnancy. Evidence from non-pregnant adult populations demonstrates that zinc supplementation reliably increases circulating zinc, with the strongest responses observed in individuals with low or depleted baseline status [33,34,35]. Notably, despite MMS supplementation, maternal zinc concentrations showed a persistent decline through pregnancy (perhaps in part due to hemodilution) and into lactation, with women failing to return to early pregnancy concentrations by 3 months postpartum. Our previous findings showed that MMS supplementation improved maternal zinc status compared to IFA at late pregnancy and in cord blood [23,24]. However, the absence of an association between maternal supplementation and infant zinc status, despite supplementation with MMS, including 12 mg zinc daily from early pregnancy to 3 months postpartum, indicates potential limitations in currently set recommendations in pregnancy and lactation for optimizing infant zinc status.

Our findings demonstrate that while milk zinc content itself was not associated with infant plasma zinc, exclusively breastfed infants showed higher zinc concentrations compared to partially breastfed infants during early infancy, a critical developmental window when endogenous zinc regulation remains immature and breastmilk serves as the primary and most bioavailable zinc source [4]. Breast milk zinc concentrations were notably higher and showed a wider distribution compared to maternal plasma zinc values—relationships shown previously and consistent with the mammary gland’s active concentration mechanisms to prioritize zinc delivery to the infant via milk [36]. Notably, the differences by feeding type emerged within a study population with a high prevalence of exclusive breastfeeding. These findings collectively emphasize the importance of sustaining exclusive breastfeeding to optimize infant zinc at 3 months of age during this foundational developmental phase.

The positive infant ferritin–zinc association, persisting after inflammation adjustment, suggests complicated metabolism despite iron-zinc competition via divalent metal transporter 1 (DMT1) during absorption [37]. Infants with higher concentrations of ferritin—a marker of iron storage—demonstrated better zinc status, suggesting adequate transfer of both nutrients through improved maternal micronutrient status that benefits the mother–infant dyad [24]. The positive association between AGP and zinc observed in our unadjusted model disappeared after adjustment for covariates, suggesting this initial relationship was driven by confounding factors rather than a true biological association. While zinc concentrations typically decrease due to tissue redistribution during inflammation [38,39] as AGP becomes elevated, the relationship between zinc and AGP was inexplicably positive in our unadjusted model.

Our findings are consistent with a role for zinc in linear growth, while an association with ponderal growth was lacking. Meta-analyses of zinc supplementation trials demonstrate modest but consistent improvements in linear growth of infants, particularly in populations with high stunting prevalence [7,40]. Limited research exists examining the relationship between infant circulating zinc concentrations and growth outcomes, though emerging evidence suggests a complex bidirectional relationship where zinc status influences growth velocity while growth itself modulates plasma zinc concentrations through increased utilization during periods of rapid development [41,42]. Zinc’s role in cellular differentiation and insulin-like growth factor-1 (IGF-1)-mediated bone growth could explain its distinct association with length gain as zinc directly regulates DNA synthesis through thymidine kinase, maintains the GH-IGF-1 axis, and enables post-receptor signal transduction [43]. The concurrent positive association with LAZs and inverse trend with WLZs suggests that zinc may support linear growth trajectories ahead of gains in soft tissue mass. The inconsistency between zinc’s linear and ponderal effects highlights the need for growth outcome specificity when evaluating nutritional interventions.

This study addresses a key gap by measuring infant plasma zinc at 3 months, an age with sparse zinc status data, and its longitudinal follow-up from early pregnancy to 3 months postpartum offers a view of zinc dynamics across the reproductive continuum. However, interpretation is limited by a modest sample size; the assessment of growth at the same time as zinc status measurement, which precludes examining subsequent growth trajectories; and measurement challenges (zinc assessed at different time points with different methods). Although sourced from a randomized trial, the present analyses are observational, so causal inference is not possible. Contextualizing our findings is further constrained by the scarcity of comparable early-infancy studies and by zinc deficiency cutoffs, which have not been validated for early infancy or the postpartum period.

This study highlights the connection between maternal biology, infant metabolism, and other factors in shaping early life zinc status. Our findings suggest that targeted interventions of zinc may need to prioritize both younger adolescent and multiparous mothers, focusing on preconception and early pregnancy zinc adequacy. Breastfeeding promotion remains critical, though strategies to enhance breastmilk zinc content or bioavailability warrant further exploration. The findings in relation to growth emphasize the need to support zinc status in early infancy, before consequences of linear growth faltering become evident.

## Figures and Tables

**Table 1:** Characteristics of participants in study of infant plasma zinc concentrations at 3 months postpartum in rural Bangladesh ^1^.

		All (*n* = 317)	Male (*n* = 179)	Female (*n* = 138)
Maternal	Age at enrollment, yrs	22.89 ± 5.35	22.82 ± 5.22	22.98 ± 5.54
	<20	96 (30.3%)	50 (27.9%)	46 (33.3%)
	20–30	185 (58.4%)	113 (63.1%)	72 (52.2%)
	≥30	36 (11.4%)	16 (8.9%)	20 (14.5%)
	Parity			
	0	115 (36.3%)	67 (37.4%)	48 (34.8%)
	1	100 (31.6%)	58 (32.4%)	42 (30.4%)
	≥2	102 (32.2%)	54 (30.2%)	48 (34.8%)
	Literacy, %	198 (62.5%)	115 (64.3%)	83 (60.1%)
	Height, cm	148.61 ± 5.19	148.78 ± 5.02	148.38 ± 5.42
	Early pregnancy weight, kg	43.74 ± 5.78	43.66 ± 5.66	43.85 ± 5.97
	Early pregnancy (baseline) zinc, µmol/L	11.00 ± 2.56	10.98 ± 2.31	11.04 ± 2.86
	Late pregnancy (third trimester) zinc, µmol/L	10.37 ± 5.16	10.52 ± 5.19	10.18 ± 5.13
	Postpartum zinc ^2^, µmol/L	9.43 ± 3.70	9.53 ± 3.59	9.31 ± 3.86
	Breastmilk zinc ^2^, µmol/L	25.05 ± 15.52	23.86 ± 12.28	26.62 ± 18.90
	Intervention (MMS) ^3^, %	162 (51.1%)	91 (50.8%)	71 (51.5%)
Infant Birth	Gestational age, wk	39.35 ± 2.43	39.40 ±2.41	39.32 ± 2.46
	Weight, kg	2.60 ± 0.04	2.63 ± 0.40	2.55 ± 0.39
	Length, cm **	46.79 ± 1.97	47.07 ± 2.00	46.43 ± 1.88
	Weight z-score	−1.60 ± 0.96	−1.60 ± 0.94	−1.59 ± 0.99
	Length z-score	−1.53 ± 1.04	−1.54 ± 1.06	−1.51 ± 1.02
	Weight-for-length z-score	−0.81 ± 1.03	−0.86 ± 1.06	−0.74 ±0.78
	LBW, %	133 (42.0%)	69 (38.6%)	64 (46.4%)
	SGA, %	207 (67.4%)	123 (70.3%)	84 (63.6%)
	Preterm,%	36 (11.7%)	21 (12.0%)	15 (11.4%)
	Cord blood zinc, µmol/L	14.76 ± 3.74	14.65 ± 3.74	14.92 ± 3.79
Infant, 3 mo	Age, day	95.44 ± 6.33	94.98 ± 5.27	96.04 ± 7.47
	Weight, kg ***	5.28 ± 0.73	5.51 ± 0.65	5.01 ± 0.72
	Length, cm ***	58.31 ± 2.30	59.1 ± 2.08	57.40 ± 2.25
	Weight z-score	−1.39 ± 1.06	−1.38 ± 0.98	−1.40 ± 1.16
	Length z-score	−1.33 ± 1.01	−1.34 ± 1.01	−1.31 ± 1.03
	Weight-for-length z-score	−0.43 ± 1.18	−0.43 ± 0.98	−0.43 ± 1.41
	Rate of weight gain ^4^, gram/d ***	28.50 ± 6.11	30.58 ± 5.01	25.80 ± 6.36
	Rate of length gain ^5^, cm/month ***	3.64 ± 0.51	3.80 ± 0.47	3.45 ± 0.48
	Breastfeeding, % **			
	Partial	22 (6.9%)	14 (7.8%)	8 (5.8%)
	Predominant	32 (10.1%)	26 (14.5%)	6 (4.4%)
	Exclusive	263 (83.0%)	139 (77.7%)	124 (89.9%)
	Ferritin, µg/L *	147.16 ± 96.76	135.24 ± 91.21	161.91 ± 101.66
	AGP, g/L	0.93 ± 0.35	0.95 ± 0.34	0.91 ± 0.36
	Zinc, µmol/L	15.34 ± 6.39	15.17 ± 6.41	15.57 ± 6.38

^1^ Maternal early pregnancy: *n* = 316; maternal late pregnancy: *n* = 315; maternal postpartum: *n* = 315; breastmilk zinc: *n* = 273; cord blood: *n* = 83; infants at 3 months: *n* = 317. ^2^ Samples were collected at 3 months postpartum and analyzed using ICP-MS. ^3^ MMS: multiple micronutrient supplementation. ^4^ Calculated by weight at 3 months minus weight at birth in grams, divided by infant age in days. ^5^ Calculated by length at 3 months minus length at birth in centimeters, divided by infant age in months (30 days). *** [0, 0.001]; ** (0.001, 0.01]; * (0.01, 0.05].

**Table 2:** Maternal and infant determinants of infant plasma zinc (µmol/L) at 3 months of age in rural Bangladesh (*n* = 236) ^1^.

		Unadjusted			Adjusted ^2^	
	β Coefficient	95% CI	*p*-Value	β Coefficient	95% CI	*p*-Value
Maternal Age (Ref: <20)						
20–30 years	0.072	−0.037, 0.182	0.195	0.110	0.019, 0.201	0.018
≥30 years	0.172	0.004, 0.340	0.045	0.278	0.094, 0.461	0.003
Parity (Ref: Nulliparous)						
Parity 1	−0.072	−0.193, 0.049	0.242	−0.060	−0.187, 0.066	0.351
Parity ≥ 2	−0.010	−0.134, 0.115	0.877	−0.131	−0.234, −0.027	0.013
Early Pregnancy Zinc (µmol/L)	0.020	0.008 0.033	0.001	0.012	0.003, 0.022	0.011
Intervention (Ref: Iron–Folic Acid)	−0.018	−0.104, 0.068	0.679	−0.054	−0.134, 0.026	0.183
Infant Age (day)	−0.002	−0.008, 0.004	0.484	0.004	−0.003, 0.010	0.275
Infant Sex (Ref: Male)	0.033	−0.066, 0.118	0.410	0.033	−0.050, 0.116	0.440
Breastfeeding Status (Ref: Exclusive)						
Predominant	−0.031	−0.203, 0.142	0.728	−0.087	−0.199, 0.026	0.132
Partial	−0.065	−0.254, 0.125	0.504	−0.151	−0.294, −0.008	0.038
Breast Milk Zinc (µmol/L)	0.049	−0.028, 0.126	0.213	0.050	−0.015, 0.115	0.130
Infant Ferritin (µg/L)	0.0005	0.0002, 0.001	0.007	0.001	0.0002, 0.001	0.007
Infant AGP ^3^ (g/L)	0.153	0.050, 0.256	0.004	0.063	−0.025, 0.150	0.160

^1^ Maternal age categories: <20 (ref), 20–30, ≥30 years. Parity: nulliparous (ref), parity 1, parity ≥ 2. Infant sex: male (ref), female. Breastfeeding status at the time of the 3-month infant blood draw: exclusive (ref), predominant, partial. Intervention: multiple micronutrient supplements vs. iron–folic acid (ref). Breast milk zinc concentrations were log-transformed prior to analysis. ^2^ Adjusted models included all variables listed in the table. ^3^ AGP, α-1-acid glycoprotein.

**Table 3:** Zinc at late pregnancy, postpartum and cord blood as determinants of infant plasma zinc at 3 months of age in rural Bangladesh ^1^.

		Unadjusted			Adjusted ^2^	
	β Coef. ^3^	95% CI	*p*-Value	β Coef. ^3^	95% CI	*p*-Value
Late Pregnancy (*n* = 238)	0.004	−0.007, 0.014	0.503	0.004	−0.007, 0.015	0.498
3 mo Postpartum (*n* = 235)	0.0002	−0.007, 0.007	0.958	0.001	−0.008, 0.010	0.772
Cord Blood (*n* = 65)	0.001	−0.024, 0.027	0.927	0.007	−0.008, 0.021	0.378

^1^ Sample sizes vary due to data availability. ^2^ Adjusted for maternal age, parity, intervention status, infant age, infant sex, breastfeeding status, breast milk zinc at 3 months, infant ferritin, and infant AGP. ^3^ Beta coefficient represents change in infant plasma zinc (µmol/L) per µmol/L increase in maternal/cord blood zinc.

**Table 4:** Association of infant anthropometric parameters at 3 months of age per 1 µmol/L increment in plasma zinc among infants in rural Bangladesh (*n* = 302) ^1^.

	Unadjusted			Adjusted ^2^		
	β Coef.	95% CI	*p*-Value	β Coef.	95% CI	*p*-Value
Length-For-Age Z-score	0.017	0.003, 0.316	0.018	0.015	0.0002, 0.030	0.047
Weight-For-Age Z-score	0.006	−0.007, 0.019	0.205	0.005	−0.010, 0.020	0.479
Weight-For-Length Z-score	−0.012	−0.027, 0.069	0.176	−0.014	−0.029, 0.001	0.060
Rate of Length Gain ^3^	0.001	−0.001, 0.004	0.336	0.006	−0.001, 0.014	0.095
Rate of Weight Gain ^4^	−0.0004	−0.003, 0.002	0.709	−0.022	−0.088, 0.044	0.521

^1^ Anthropometric Z-scores were derived using the WHO Child Growth Standards. ^2^ Adjusted models of Z-scores controlled for breastfeeding status, gestational age, and intervention; adjusted models of rate of length/weight gain controlled for infant sex, gestational age, breastfeeding status at 3 months, intervention, and birth length and birth weight adjusted only in their corresponding models. ^3^ Calculated by length at 3 months minus length at birth in centimeters, divided by infant age in months (30 days). ^4^ Calculated by weight at 3 months minus weight at birth in grams, divided by infant age in days.

## Data Availability

The data presented in this study are available on request from the corresponding author. Public posting is not permitted under the terms of the funding agreement with the Bill & Melinda Gates Foundation and the study’s ethics approvals.

## Supplementary Materials

The following supporting information can be downloaded at: https://www.mdpi.com/article/10.3390/nu17213393/s1, Supplementary Table S1: Characteristics of participants in the main study and substudy of JiVitA-3 trial in rural Bangladesh.

## Abbreviations

AGP, α1-acid glycoprotein; DMT1, divalent metal transporter 1; GEEs, generalized estimating equations; IFA, iron–folic acid; IGF1, insulin-like growth factor 1; IZiNCG, International Zinc Nutrition Consultative Group; JIMMI Study, JiVitA-3 Iron-Folic Acid versus Multiple Micronutrients in Milk for Infants Study; LAZ, length-for-age z-score; LBW, low birth weight; MMS, multiple micronutrient supplementation; NIST SRM (National Institute of Standards and Technology Standard Reference Material); SGA, small for gestational age; WAZ, weight-for-age z-score; WHO, World Health Organization; WLZ, weight-for-length z-score.

## References

[1] Prasad A.S. (2013). Discovery of human zinc deficiency: Its impact on human health and disease. Adv. Nutr..

[2] Roohani N., Hurrell R., Kelishadi R., Schulin R. (2013). Zinc and its importance for human health: An integrative review. J. Res. Med. Sci..

[3] Cusick S.E., Georgieff M.K. (2016). The Role of Nutrition in Brain Development: The Golden Opportunity of the “First 1000 Days”. J. Pediatr..

[4] Krebs N.F., Miller L.V., Hambidge K.M. (2014). Zinc deficiency in infants and children: A review of its complex and synergistic interactions. Paediatr. Int. Child. Health.

[5] Terrin G., Berni Canani R., Di Chiara M., Pietravalle A., Aleandri V., Conte F., De Curtis M. (2015). Zinc in Early Life: A Key Element in the Fetus and Preterm Neonate. Nutrients.

[6] Black R.E., Victora C.G., Walker S.P., Bhutta Z.A., Christian P., de Onis M., Ezzati M., Grantham-McGregor S., Katz J., Martorell R. (2013). Maternal and child undernutrition and overweight in low-income and middle-income countries. Lancet.

[7] Imdad A., Rogner J., Sherwani R.N., Sidhu J., Regan A., Haykal M.R., Tsistinas O., Smith A., Chan X.H.S., Mayo-Wilson E. (2023). Zinc supplementation for preventing mortality, morbidity, and growth failure in children aged 6 months to 12 years. Cochrane Database Syst. Rev..

[8] Gibson R.S., Raboy V., King J.C. (2018). Implications of phytate in plant-based foods for iron and zinc bioavailability, setting dietary requirements, and formulating programs and policies. Nutr. Rev..

[9] Akhtar S. (2013). Zinc status in South Asian populations--an update. J. Health Popul. Nutr..

[10] Ahmed F., Prendiville N., Narayan A. (2016). Micronutrient deficiencies among children and women in Bangladesh: Progress and challenges. J. Nutr. Sci..

[11] Institute of Public Health Nutrition, United Nations Children’s Fund (UNICEF), Global Alliance for Improved Nutrition, International Centre for Diarrhoeal Disease Research, Bangladesh, Dhaka (ICDDR,B) (2013). National Micronutrients Status Survey 2011–12: Final Report.

[12] Hotz C., Peerson J.M., Brown K.H. (2003). Suggested lower cutoffs of serum zinc concentrations for assessing zinc status: Reanalysis of the second National Health and Nutrition Examination Survey data (1976-1980). Am. J. Clin. Nutr..

[13] Brown K.H., Rivera J.A., Bhutta Z., Gibson R.S., King J.C., Lönnerdal B., Ruel M.T., Sandtröm B., Wasantwisut E., Hotz C. (2004). International Zinc Nutrition Consultative Group (IZiNCG) technical document #1. Assessment of the risk of zinc deficiency in populations and options for its control. Food Nutr. Bull..

[14] King J.C. (2000). Determinants of maternal zinc status during pregnancy. Am. J. Clin. Nutr..

[15] Kambe T., Tsuji T., Hashimoto A., Itsumura N. (2015). The Physiological, Biochemical, and Molecular Roles of Zinc Transporters in Zinc Homeostasis and Metabolism. Physiol. Rev..

[16] McCormick N.H., Hennigar S.R., Kiselyov K., Kelleher S.L. (2014). The biology of zinc transport in mammary epithelial cells: Implications for mammary gland development, lactation, and involution. J. Mammary Gland. Biol. Neoplasia.

[17] Dror D.K., Allen L.H. (2018). Overview of Nutrients in Human Milk. Adv. Nutr..

[18] Eneroth H., El Arifeen S., Persson L.A., Kabir I., Lönnerdal B., Hossain M.B., Ekström E.C. (2009). Duration of exclusive breast-feeding and infant iron and zinc status in rural Bangladesh. J. Nutr..

[19] Osendarp S.J., Santosham M., Black R.E., Wahed M.A., van Raaij J.M., Fuchs G.J. (2002). Effect of zinc supplementation between 1 and 6 mo of life on growth and morbidity of Bangladeshi infants in urban slums. Am. J. Clin. Nutr..

[20] Elizabeth K.E., Krishnan V., Vijayakumar T. (2008). Umbilical cord blood nutrients in low birth weight babies in relation to birth weight & gestational age. Indian. J. Med. Res..

[21] Jyotsna S., Amit A., Kumar A. (2015). Study of serum zinc in low birth weight neonates and its relation with maternal zinc. J. Clin. Diagn. Res..

[22] West K.P., Shamim A.A., Mehra S., Labrique A.B., Ali H., Shaikh S., Klemm R.D., Wu L.S., Mitra M., Haque R. (2014). Effect of maternal multiple micronutrient vs iron-folic acid supplementation on infant mortality and adverse birth outcomes in rural Bangladesh: The JiVitA-3 randomized trial. JAMA.

[23] Schulze K.J., Mehra S., Shaikh S., Ali H., Shamim A.A., Wu L.S., Mitra M., Arguello M.A., Kmush B., Sungpuag P. (2019). Antenatal Multiple Micronutrient Supplementation Compared to Iron-Folic Acid Affects Micronutrient Status but Does Not Eliminate Deficiencies in a Randomized Controlled Trial Among Pregnant Women of Rural Bangladesh. J. Nutr..

[24] Schulze K.J., Gernand A.D., Khan A.Z., Wu L.S., Mehra S., Shaikh S., Ali H., Shamim A.A., Sungpuag P., Udomkesmalee E. (2020). Newborn micronutrient status biomarkers in a cluster-randomized trial of antenatal multiple micronutrient compared with iron folic acid supplementation in rural Bangladesh. Am. J. Clin. Nutr..

[25] (2024). Inorganic Constituents in Animal Serum.

[26] Nóbrega J.A., Santos M.C., de Sousa R.A., Cadore S., Barnes R.M., Tatro M. (2006). Sample preparation in alkaline media. Spectrochim. Acta Part. B At. Spectrosc..

[27] Lee J., Park Y.-S., Lee H.-J., Koo Y.E. (2022). Microwave-assisted digestion method using diluted nitric acid and hydrogen peroxide for the determination of major and minor elements in milk samples by ICP-OES and ICP-MS. Food Chem..

[28] (2025). Organic Contaminants in Fortified Human Milk.

[29] (2018). Infant/Adult Nutritional Formula I (Milk-Based).

[30] World Health Organization WHO Child Growth Standards. https://www.who.int/tools/child-growth-standards.

[31] Islam M.R., Rahman S.M., Tarafder C., Rahman M.M., Rahman A., Ekström E.-C. (2020). Exploring Rural Adolescents’ Dietary Diversity and Its Socioeconomic Correlates: A Cross-Sectional Study from Matlab, Bangladesh. Nutrients.

[32] Nguyen P.H., Huybregts L., Sanghvi T.G., Tran L.M., Frongillo E.A., Menon P., Ruel M.T. (2018). Dietary Diversity Predicts the Adequacy of Micronutrient Intake in Pregnant Adolescent Girls and Women in Bangladesh, but Use of the 5-Group Cutoff Poorly Identifies Individuals with Inadequate Intake. J. Nutr..

[33] Hess S.Y., Peerson J.M., King J.C., Brown K.H. (2007). Use of serum zinc concentration as an indicator of population zinc status. Food Nutr. Bull..

[34] Lowe N.M., Medina M.W., Stammers A.L., Patel S., Souverein O.W., Dullemeijer C., Serra-Majem L., Nissensohn M., Hall Moran V. (2012). The relationship between zinc intake and serum/plasma zinc concentration in adults: A systematic review and dose-response meta-analysis by the EURRECA Network. Br. J. Nutr..

[35] Wieringa F.T., Dijkhuizen M.A., Fiorentino M., Laillou A., Berger J. (2015). Determination of zinc status in humans: Which indicator should we use?. Nutrients.

[36] Donangelo C.M., King J.C. (2012). Maternal Zinc Intakes and Homeostatic Adjustments during Pregnancy and Lactation. Nutrients.

[37] Whittaker P. (1998). Iron and zinc interactions in humans. Am. J. Clin. Nutr..

[38] Hochepied T., Berger F.G., Baumann H., Libert C. (2003). Alpha(1)-acid glycoprotein: An acute phase protein with inflammatory and immunomodulating properties. Cytokine Growth Factor. Rev..

[39] McDonald C.M., Suchdev P.S., Krebs N.F., Hess S.Y., Wessells K.R., Ismaily S., Rahman S., Wieringa F.T., Williams A.M., Brown K.H. (2020). Adjusting plasma or serum zinc concentrations for inflammation: Biomarkers Reflecting Inflammation and Nutritional Determinants of Anemia (BRINDA) project. Am. J. Clin. Nutr..

[40] Brown K.H., Peerson J.M., Rivera J., Allen L.H. (2002). Effect of supplemental zinc on the growth and serum zinc concentrations of prepubertal children: A meta-analysis of randomized controlled trials. Am. J. Clin. Nutr..

[41] Altigani M., Murphy J.F., Gray O.P. (1989). Plasma zinc concentration and catch up growth in preterm infants. Acta Paediatr. Scand. Suppl..

[42] Wastney M.E., Long J.M., McDonald C.M., Krebs N.F., Islam M.M., Ahmed T., Khandaker A.M., Sthity R.A., Westcott J.E., King J.C. (2022). Zinc Kinetics Correlate With Length-for-Age z Scores in Bangladeshi Infants. J. Pediatr. Gastroenterol. Nutr..

[43] MacDonald R.S. (2000). The Role of Zinc in Growth and Cell Proliferation. J. Nutr..

