# Supplementary material for: Maternal and Infant Determinants of Zinc Status and Zinc’s Association with Anthropometry in 3-Month-Old Bangladeshi Infants
Source: Nutrients. 2025 Oct 29;17(21):3393. doi: 10.3390/nu17213393 (PMC12610514; doi:10.3390/nu17213393)
Supplement: Supplementary file 1 [file nutrients-17-03393-s001.zip › nutrients-3948737-supplementary.pdf]

Supplementary Table 1: Characteristics of Participants in the Main Study and Sub-study of JiVitA-3 trail in Rural Bangladesh[1]

		All (n=752)	Excluded: No Zinc Data (n=435)	Study Sample (n=317)
**Maternal Baseline Characteristics**	Age at enrollment, *yrs*	22.92 ± 5.37	22.94 ± 5.40	22.89 ± 5.35
	Gestational age, *wks*	39.34 ± 2.41	39.33 ± 2.40	39.36 ± 2.43
	Parity<br>0<br>1<br>>2	 267 (35.5%)<br>247 (32.9%)<br>238 (31.6%)	 152 (34.9%)<br>147 (33.8%)<br>136 (31.3%)	 115 (36.3%)<br>100 (31.5%)<br>102 (32.2%)
	Literacy, %	479 (63.7%)	281 (64.6%)	198 (62.5%)
	Height, *cm*	148.94 ± 5.18	148.92 ± 5.17	148.98 ± 5.19
	Early pregnancy weight, *kg*	42.96 ± 6.08	42.84 ± 5.92	43.12 ± 6.29
	Late pregnancy weight, *kg*	48.44 ± 6.09	48.35 ± 6.05	48.56 ± 6.15
	Early pregnancy BMI, kg/m^2	19.33 ± 2.29	19.29 ± 2.21	19.40 ± 2.39
	LSI	-0.03 ± 0.95	-0.07 ± 0.95	0.02 ± 0.95
	Season of blood draw[2]<br>Hot-dry<br>Monsoon<br>Winter	 305 (40.6%)<br>201 (26.7%)<br>246 (32.7%)	 178 (40.9%)<br>122 (28.1%)<br>135(31.0%)	 127 (40.1%)<br>79 (24.9%)<br>111 (35.0%)
**Maternal Baseline Diet[3]**	Meat<br>≥ 3	 105 (14.0%)	 41 (9.4%)	 61 (19.2%)
	Fish<br>≥ 3	 473 (62.9%)	 267 (61.4%)	 206 (65.0%)
	Milk<br>≥ 3	 178 (23.7%)	 109 (25.1%)	 69 (21.8%)
	Egg<br>≥ 3	 129 (17.2%)	 72 (16.6%)	 57 (18.0%)
	Yellow vegetable<br>< 3<br>≥ 3	 629 (83.6%)<br>123 (16.4%)	 354 (81.4%)<br>81 (18.6%)	 275 (86.8%)<br>42 (13.2%)
	Dark leafy vegetable<br>≥ 3	 184 (24.5%)	 112 (25.7%)	 72 (22.7%)
**Maternal Baseline Morbidity[4]**	Nausea	365 (48.5%)	213 (49.0%)	152 (48.0%)
	Vomiting	177 (23.5%)	110 (25.3%)	67 (21.1%)
	Low fever	243 (32.3%)	148 (34.0%)	95 (30.0%)
	High fever	10 (1.3%)	5 (1.2%)	5 (1.6%)
	Cough	109 (14.5%)	60 (13.8%)	49 (15.5%)
**Infant Birth**	Gender, male %	395 (52.5%)	216 (49.7%)	179 (56.5%)
	Weight, *kg*	2.59 ± 0.39	2.58 ± 0.39	2.60 ± 0.40
	Length*, cm*	46.8 ± 2.02	46.73 ± 2.06	46.79 ± 1.97
	Weight z-score	-1.60 ± 0.96	-1.61 ± 0.97	-1.60 ± 0.96
	Length z-score	-1.53 ± 1.08	-1.53 ± 1.11	-1.53 ± 1.04
	Weight/length z-score	-0.79 ±1.08	-0.78 ± 1.02	-0.81 ±1.03
	LBW, %	311 (41.4%)	178 (40.9%)	133 (42.0%)

	SGA, %	365 (54.7%)	212 (54.8%)	153 (54.5%)
	Preterm, %	83 (11.5%)	47 (11.3%)	36 (11.7%)
	Breastfeeding, %<br>Partial<br>Predominant<br>Exclusive	 21 (2.8%)<br>226 (30.1%)<br>505 (67.2%)	 13 (3.0%)<br>130 (29.9%)<br>292 (67.1%)	 8 (2.5%)<br>96 (30.3%)<br>213 (67.2%)
**Infant at 3 months Characteristics**	Age, days	95.01 ± 5.72	94.69 ± 5.22	95.44 ± 6.33
	Weight, *kg*	5.26 ± 0.71	5.23 ± 0.70	5.30 ± 0.73
	Length, *cm*	58.18 ± 2.36	58.09 ± 2.41	58.31 ± 2.30
	Weight z-score	-1.41 ± 1.05	-1.42 ± 1.05	-1.39 ± 1.01
	Length z-score	-1.34 ± 1.07	-1.35 ± 1.12	-1.33 ±1.01
	Weight/length z-score	-0.43 ± 1.19	-0.42 ±1.20	-0.43 ± 1.18
	Breastfeeding, %<br>Partial<br>Predominant<br>Exclusive	 57 (7.6%)<br>54 (7.2%)<br>641 (85.2%)	 35 (8.1%)<br>22 (5.1%)<br>378 (86.9%)	 22 (6.9 %)<br>32 (10.1%)<br>263 (83.0%)
	Breastfeeding frequency<br>1-10 times<br>11-20 times<br>>20 times	 55 (7.3%)<br>510 (67.8%)<br>187 (24.9%)	 31 (7.1%)<br>301 (69.2%)<br>103 (23.7%)	 24 (7.6%)<br>209 (65.9%)<br>84 (26.5%)
	Breastmilk sufficiency<br>No<br>Yes	 76 (10.1%)<br>676 (89.9%)	 49 (11.3%)<br>386 (88.7%)	 27 (8.5%)<br>290 (91.5%)
**Infant at 3 months Morbidity[4]**	Cough	458 (68.9%)	265 (69.4%)	193 (68.2%)
	Fever	73 (26.0%)	39 (23.5%)	34 (29.6%)
**Maternal 3MPP Characteristics**	Height, *cm*	148.65 ± 5.18	148.69 ± 5.17	148.61 ± 5.19
	Weight, *kg*	43.7 ± 5.72	43.69 ± 5.68	43.74 ± 5.79
	BMI, *kg/m²*	19.75 ± 2.09	19.73 ± 2.01	19.78 ± 2.20
	Season of blood draw[2]<br>Hot-dry<br>Monsoon<br>Winter	 253 (33.6%)<br>211 (28.1%)<br>288 (38.3%)	 158 (36.3%)<br>113 (26.0%)<br>164 (37.7%)	 95 (30.0%)<br>98 (30.9%)<br>124 (39.1%)
**Diet, Maternal 3MPP[3]**	Meat<br>≥ 3	 101 (13.4)	 62 (14.3%)	 39 (12.3%)
	Fish<br>≥ 3	 473 (62.9%)	 275 (63.2)	 198 (62.5%)
	Milk<br>≥ 3	 117 (15.6%)	 77 (17.7%)	 40 (12.6%)
	Egg<br>≥ 3	 58 (7.7%)	 36 (8.3%)	 22 (6.9%)
	Yellow vegetable<br>≥ 3	 81 (10.8%)	 54 (12.4%)	 27 (8.5%)
	Dark leafy vegetable<br>≥ 3	 74 (9.8%)	 42 (9.7%)	 32 (10.1%)

[1]Values are mean ± SD or n(%); BMI, body mass index; LSI, living standard index; 3MPP, 3-month postpartum; LBW, low birth weight; SGA, small for gestational age.
[2]Hot-dry, February 16th to June 15th; monsoon, Jun 16th to October 15th; winter, October 16th to February 15th.
[3]Diet is documented as consumed < 3 or ≥ 3 times in the last week.
[4]Morbidity is reported as symptom present ≥ 1 day in the last week
